# Healthcare Professionals’ Knowledge about Pediatric Chronic Pain: A Systematic Review

**DOI:** 10.3390/children10040665

**Published:** 2023-03-31

**Authors:** Mónica Pico, Carmen Matey-Rodríguez, Ana Domínguez-García, Héctor Menéndez, Simone Lista, Alejandro Santos-Lozano

**Affiliations:** 1 i+HeALTH Strategic Research Group, Department of Health Sciences, Miguel de Cervantes European University (UEMC), 47012 Valladolid, Spainasantos@uemc.es (A.S.-L.); 2 Research Institute of the Hospital 12 de Octubre (‘imas12’), 28041 Madrid, Spain

**Keywords:** child, chronic pain, health knowledge attitudes practice, pediatrics

## Abstract

Pediatric chronic pain is a common public health problem with a high prevalence among children and adolescents. The aim of this study was to review the current knowledge of health professionals on pediatric chronic pain between 15–30% among children and adolescents. However, since this is an underdiagnosed condition, it is inadequately treated by health professionals. To this aim, a systematic review was carried out based on a search of the electronic literature databases (PubMed and Web of Science), resulting in 14 articles that met the inclusion criteria. The analysis of these articles seems to show a certain degree of heterogeneity in the surveyed professionals about the awareness of this concept, especially regarding its etiology, assessment, and management. In addition, the extent of knowledge of the health professionals seems to be insufficient regarding these aspects of pediatric chronic pain. Hence, the knowledge of the health professionals is unrelated to recent research that identifies central hyperexcitability as the primary factor affecting the onset, persistence, and management of pediatric chronic pain.

## 1. Introduction

Pain is a public health problem and the most frequent reason leading individuals to seek medical attention [1,2]. During the last decade, the prevalence of individuals having chronic or recurrent pain—persisting or recurring for more than 3 months [3]—increased; actually, around 15–30% of children and adolescents are estimated to experience it [4,5,6,7,8,9].

Although the etiology of pediatric chronic pain is not clear [10], its study is currently focused on two dimensions: the physical and the psychosocial condition. Primary physical causes are medical pathologies, invasive procedures, physical injuries, and other unknown factors [11,12]. Among the psychological ones, research has highlighted that tension, stress, and other situations where self-esteem is damaged can cause pain and somatization of symptoms [13,14]. There are also risk factors associated with an increased likelihood of chronic pain, such as lifestyle (diet, hydration levels, tobacco, and alcohol consumption—in adolescents—or physical inactivity) [15] or family context [16,17,18,19]. In addition, somatic, psychological, and social factors act as modulators of pain [20].

Pediatric chronic pain is one of the most underdiagnosed and, consequently, undertreated clinical aspects [21,22,23]. The traditional approach adopted a dualistic viewpoint that conceptualized the mind and body as functioning separately and independently [24]. The main approach undertaken has been based on a bio-psychosocial model in healthcare, in the last decades [24,25,26], which considers that chronic pain is explained by biological, social, and psychological reasons [24], prioritizing management from a multidisciplinary perspective [27,28].

For patients, this reconceptualization of chronic pain as multifactorial can produce positive effects, such as improving pain and pain-related disability [20]. However, such an approach is not currently managed in the most appropriate way [23] because of (I) difficulties met by healthcare professionals in the assessment and management of pain and (II) incorrect attitudes and beliefs about its origin [29,30]. This study aims to provide a review of the current knowledge of healthcare professionals concerning pediatric chronic pain, primarily in terms of its assessment and management.

## 2. Materials and Methods

### 2.1. Protocol Registration

This review is registered in the PROSPERO international database (https://www.crd.york.ac.uk/prospero/display_record.php?ID=CRD42021205588) (accessed on 14 April 2022) and follows the PRISMA recommendations for systematic reviews [31].

### 2.2. Eligibility Criteria

The criteria for inclusion of articles were as follows: (1) participants had to include healthcare professionals with university degrees who work directly with children diagnosed with chronic or recurrent pain, (2) articles had to report on the knowledge of healthcare professionals mentioned above, and (3) articles had to be cross-sectional studies without intervention.

Articles where patients had acute, procedural, palliative, oncologic, disability-related, or preterm infant pain were excluded. Articles not written in English or Spanish were excluded.

### 2.3. Sources Information and Search

A systematic search of the existing literature was carried out using the electronic literature PubMed and Web of Science databases on 15 October 2022. The search box used in both databases was as follows: (“pain”) AND (pediatric OR child OR children) AND (knowledge OR attitudes OR attitude OR beliefs OR belief OR education OR “pain education” OR assessment OR management) AND (pediatrician OR pediatricians OR nurses OR “health professionals” OR care professionals OR “pediatric nurses”)”.

### 2.4. Data Extraction Process and Data List

Data were extracted by two authors (MP, AD) and collected in an Excel spreadsheet. Potential disagreements were solved by a third author (ASL). The information extracted from each article were as follows: (1) article data; (2) sociodemographic features of the sample; (3) type of pediatric chronic pain; and (4) pediatricians’ knowledge of etiology, prevalence, assessment, management, and consequences of pediatric chronic pain.

### 2.5. Study Selection

Before screening, search results were imported into Excel and duplicates were removed. Eligibility screening was performed in duplicate, i.e., screened twice by independent reviewers (MP and CMR). Disagreements were solved through discussion or, if not possible, by a third party (AD or ASL). In a first step, screening was performed based on the study title and abstract. For studies conforming with the eligibility criteria at this stage and not meeting any of the exclusion criteria, full-text publications were accessed and again screened in duplicate.

### 2.6. Data Collection Process

Like the screening process, the data extraction required a minimum of two independent reviewers. Discrepancies were solved through discussion and involved a third party, if necessary. Missing data were recorded as not reported. Where reports were considered ambiguous, authors were contacted for clarification.

### 2.7. Data Elements

The domains of data extraction related to health professionals’ knowledge of pediatric chronic pain were etiology, assessment, management, and consequences of pediatric chronic pain. Data were also collected on healthcare workers’ self-perception of their own knowledge and their sociodemographic characteristics.

### 2.8. Risk of Bias

The quality of the studies was assessed using the tool for the systematic and qualitative investigation of studies with disparate data [32].

## 3. Results

### 3.1. Selection of Studies

**Figure 1:**
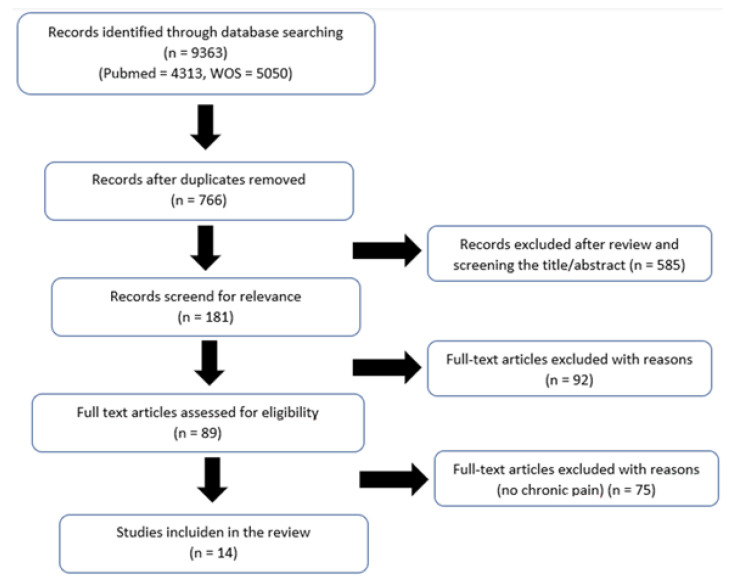
Systematic review flowchart.

The selection process of relevant articles is presented in Figure 1. The initial search conducted in PubMed and Web of Science databases led to the collection of 9363 articles. After the removal of duplicates, the remaining articles were 766. These articles were then screened by title and abstract and inclusion/exclusion criteria were applied, thus collecting 181 articles for full-text selection. In a first step, 92 articles were excluded. These included the following: (I) articles that were not cross-sectional studies and (II) articles where the healthcare professionals were students. Finally, although we found articles mentioning pediatric chronic pain, these were excluded since they referred to pain due to palliative care or oncologic processes. After the final screening, 14 articles remained available for inclusion.

### 3.2. Characteristics of the Studies Included in the Systematic Review

The characteristics of the 14 selected articles are presented in Supplementary Table S1. All studies conducted are cross-sectional; the analysis by Høie et al. [5] is a qualitative study with an exploratory design. All studies were conducted in the U.S., Australia, New Zealand, the United Kingdom, Germany, Norway, Spain, Sweden, Switzerland, and Saudi Arabia. All the articles have a high methodological quality (Table 1).

The sample of the 14 studies included 3579 healthcare professionals (see Table 2). The mean age of the participants was in the 29–70 years old range (five articles did not specify participants’ age) [33,34,35,36]; 55.9% of them were women (two articles did not specify participants’ sex) [33,36]. In the articles in which race was mentioned [37,38], most of the healthcare professionals were of Caucasian race. The professional experience ranged from 11 to 30 years. Most of the healthcare providers mentioned in the studies were pediatricians [33,35,37,38,39,40,41,42,43,44], although two studies exclusively reported nurses as professionals [5,34]. Riaño et al. [42] also included pediatric surgeons and pediatric resident doctors. Al-Khotani et al. [45] focused on pediatricians and orofacial specialists, and Miró et al. [35], in addition to pediatricians, included general practitioners. Bhatia et al. [36] mentioned pain clinicians and general practitioners. Glazebrook et al. [44] included pediatricians, nurses, physiotherapists, and occupational therapists.

**Table 1:** Risk of bias of the clinical studies included in the systematic revision.

Author	1. Abstract and Title	2. Introduction and Aims	3. Method and Data	4. Sampling	5. Data Analysis	6. Ethics and Bias	7. Findings/ Results	8. Transferability/ Generalizability	9. Implications and Usefulness	TOTAL	DEGREE
Al-Khotani et al. (2015) [45]	4	4	4	4	3	3	4	4	4	35	A
Bhatia et al. (2008) [36]	4	4	4	2	4	3	4	4	4	33	A
Edwards et al. (1994) [41]	4	4	4	4	4	4	4	3	4	35	A
Glazebrook et al. (2009) [44]	4	4	4	3	4	4	4	4	4	35	A
Heinsch et al. (2019) [39]	4	4	3	3	4	3	4	4	4	33	A
Høie et al. (2017) [5]	4	4	3	3	4	4	4	3	4	33	A
Koechlin et al. (2022) [43]	4	4	4	4	4	4	4	4	4	36	A
Miro et al. (2020) [35]	4	4	4	2	4	4	4	4	4	34	A
Riaño et al. (1998) [42]	4	4	3	4	4	1	4	4	4	32	A
Sawni et al. (2007) [37]	4	4	3	4	4	4	4	4	4	35	A
Schlarb et al. (2011) [40]	4	3	3	3	3	3	4	4	4	31	A
Schurman et al. (2014) [33]	4	4	4	3	3	4	4	4	4	34	A
Thompson et al. (2010) [38]	4	4	4	4	4	4	4	3	4	35	A
Youssef et al. (2007) [34]	4	4	3	2	4	1	4	4	4	30	A

Abbreviations: A: high quality (30–36 points).

Although the working place was not described in all the studies, some of them included a description of it [5,33,37,38,39,41,42,43]. It should be noted that the majority were healthcare professionals in the public sector, in a hospital context, located in urban areas. The questionnaire used in each study is shown in Table 3.

### 3.3. Consequences of Pediatric Chronic Pain 

Several authors [33,36,39] mentioned the negative consequences of pediatric chronic pain for the development of the child (lower quality of life and a source of stress for the family). Moreover, Youssef et al. [34] pointed out a greater school absence rate of children with chronic pain but without associated comorbidity, and almost 90% of those surveyed by Bhatia et al. [36], in addition to school absence, pointed out sleep disruption, reduced socialization, and inability to do sports as functional disabilities associated to children with chronic pain.

### 3.4. Etiology

Several investigations addressed generalized pediatric chronic pain in their research [5,35,36,37,38,42,44]. The nurses interviewed by Høie et al. [5] associated pediatric chronic pain with high academic expectations, low-stress tolerance, lifestyle, and social relationships. The healthcare providers discussed by Glazebrook et al. [44] provided a substantial emphasis on the psychosocial aspects of pediatric chronic pain, in particular, on the role of the family.

Regarding chronic pain caused by gastrointestinal disorders, most of the authors agree that the responsible cause is still under debate, although all of them indicate that pain, in this type of pathology, presents an important psychological component. This is the case of the study by Heinsch et al. [39], in which 66% of respondents thought that “the cause was is a psychological rather than a medical problem”, and that of Youssef et al. [34], in which only 3.6% of respondents believed that recurrent abdominal pain is a disease. The percent of school nurses in this study who thought that students with recurrent abdominal pain were faking was 47%; those who thought they were seeking attention was 61%, that they were nervous 31%, that they were sad 29%, or that they were lazy 78%. Only 25% thought that children with recurrent abdominal pain needed medication, and 52% thought they needed to relax more. Similar data have been found in the investigation by Edwards and colleagues [41], where respondents stated that pain had almost entirely psychological bases. Schurman et al. [33] also concluded that most of their respondents thought that multiple psychosocial problems were associated with chronic abdominal pain (anxiety, family conflict, school avoidance, depression, social problems, and academic difficulties).

Al-Khotani et al. [45] studied the knowledge of pediatric chronic pain related to orofacial pain and temporomandibular disorders. For the Swedish reference group, pediatric chronic pain etiology had somatic, behavioral, and social causes, and depression was an important etiological factor. The survey results stated that Swedish pediatricians and maxillofacial surgeons disagreed the least with this reference group. Other healthcare professionals, interviewed from Sweden and Saudi Arabia, disagreed regarding the etiology of this type of pain.

### 3.5. Assessment

Regarding the assessment of non-specific chronic pain, 97% of those surveyed by Riaño et al. [42] stressed the importance of its evaluation, although only 35% stated that they knew some assessment methods and, of these, 82% had never applied them. No differences were observed in terms of sex or age, except in the knowledge of pain assessment tools; the younger the age, the easier it is for them to know about the tools (24% of pediatricians over 45 years old knew assessment methods compared to 40% of the younger group). In the study by Miró et al. [35], 84.8% of the participants assessed pediatric chronic pain on a regular basis. The most frequently assessed domains were pain intensity (80%), treatment side-effects (67%), physical, emotional, and social function of the child (69%), and overall satisfaction with treatment (60%). The least evaluated domains were fatigue (42%), sleep (61%), and social function (60%). The reasons for not executing pediatric chronic pain assessment were lack of time and the absence of an assessment protocol. Regarding the pain assessment tools used, two studies named them [38,43]. In the study by Thompson et al. [38], most pediatricians used parent report (87.1%) followed by patient self-report (84.2%), visual or numerical pain rating scales (55.5%), nonverbal scales (66.7%), and the pain diary (49.5%). In the study by Koechlin et al. [43], most pediatricians used a subjective assessment to measure pain intensity (subjective assessment of the child’s face or behavior in 62.5% of respondents and subjective assessment based on anamnesis and examination in 72.2%).

In the case of chronic pain generated by gastrointestinal disorders, the respondents of Heinsch et al. [39] and Schurman et al. [33] used the Rome III criteria for its diagnosis. These criteria were known by 37.2% of the respondents in the study by Heinsch et al. [39], and their use was mainly associated with the outpatient setting. The research by Schurman et al. [33] showed similar data, with 42% of the respondents being aware of these criteria, although only 7% stated they used them in their practice. Heinsch et al. [39] concluded that respondents aware of the criteria were more confident in making the diagnosis of functional abdominal pain and irritable bowel syndrome (pathologies associated with gastrointestinal disorders generated pediatric chronic pain) and requested fewer tests. According to Youssef et al. [34], 31% of respondents did not know whether recurrent abdominal pain was a serious or minor condition, regardless of their years of work experience, and only 3.6% of respondents believed that recurrent abdominal pain is a disease, even stating that children may fake symptoms to attract attention. This attitude also appears in the pediatricians surveyed by Schurman et al. [33], where only 3% thought that recurrent abdominal pain was a real abdominal pain. All authors addressing the issue of pediatric chronic pain generated by gastrointestinal disorders agree on the need to request complementary tests to rule out an organic cause. This request is more than 70% according to Heinsch et al. [39] and Youssef et al. [34] and 35.6% according to Schlarb et al. [40]; it is more frequent among younger respondents and with little probability of making a diagnosis from them, according to Heinsch et al. [39]. Respondents from Heinsch et al. [39], Edwards et al. [41], and Høie et al. [5] acknowledged being influenced by parental pressure to request further diagnostic testing.

### 3.6. Management

According to Riaño et al. [42], respondents considered it essential to reduce and avoid pain in the child (85% of female pediatricians versus 69% of male pediatricians); however, 42% stated that they did not know any guidelines for doing so (the younger the health professionals are, the more guidelines they know). A calculated 55% acknowledged satisfactory pain care, compared to 42% who did poorly or unsatisfactorily, with difficulties in reliably following up in 64% of cases. Respondents surveyed by Bhatia et al. [36] stated that they lacked adequate resources for the management of chronic pain in children (83% of pain physicians and 55% of general practitioners).

In the study by Miró et al. [35], 96% of family physicians and pediatricians emphasized the need for a pediatric chronic pain management protocol, although only 3.7% stated that they always or almost always used it (14.7%). The main reasons for not using protocols were that they did not have and/or did not know where to find them, perceived lack of usefulness in clinical practice, and lack of time to implement them.

According to Heinsch et al. [39], the majority of respondents (59.8% for inflammatory bowel syndrome and 65.6% for functional abdominal pain) believed that there was a lack of clear evidence for effective therapy for chronic pain due to gastrointestinal disorders and also a lack of access to these evidence-based therapies. The knowledge about the evidence of recurrent abdominal pain and efficacy did not affect the management options selected. The main management options emerging in the studies are described below (Table 4).

#### 3.6.1. Behavioral Treatment

Eighty-two percent (82%) of the pediatricians surveyed by Schurman et al. [33] used both behavioral and medical treatments to relieve pain in children with recurrent abdominal pain, and most recommended a lifestyle change (74%) associated with reassurance (61%). According to Heinsh et al. [39], written information was the most commonly used tool, especially in young public workers for gastrointestinal disorders treatment. Schlarb et al. [40] agree with these data, with advice, support, and reassurance being the management option most used (20.1%). Respondents from Edwards et al. [41] also answered that the best way to manage was to inform both the parents and the child that everything was normal.

#### 3.6.2. Symptomatic Treatment

According to Bhatia et al. [36], the most common intervention in the management of children with chronic pain was represented by pharmacotherapy. The most used pharmacological treatments, according to Riaño et al. [42], were acetylsalicylic acid (12%), paracetamol (18%), paracetamol with codeine (37%), Metamizole (dipyrone) (28%), non-steroidal anti-inflammatory (24%), opiates (30%), and tricyclic antidepressants (3%) without observing differences between age groups. According to Thomson et al. [38], 66.9% of the pediatricians recommended non-steroidal anti-inflammatory treatment and 61.7% acetaminophen. In the study by Høie et al. [5], school nurses stated that adolescents consumed analgesics, such as acetaminophen, on a regular basis.

Schlarb et al. [40] mentioned symptomatic pain treatment with physiotherapy and pharmacology in 11.1% of the interviewees. Most of the physicians interviewed by Bhatia et al. [36] also used a combination of three or more modalities, with pharmacotherapy being the core treatment.

#### 3.6.3. Complementary Therapies

The study by Sawni et al. [37] is the only one that exclusively addresses pediatricians’ knowledge about using complementary therapies as an alternative to conventional treatments for pediatric chronic pain. The complementary therapies that were assumed to be safe were similar to those that were considered effective and included acupuncture, biofeedback, massage, relaxation, healing prayer, aromatherapy, and hypnosis. The therapies considered most harmful were herbs (61%), megavitamins (57%), chiropractic manipulation (57%), and homeopathy (23%). In this study, 96% of respondents believed that their patients used some form of CT, 71% stated that they would consider referring patients to CT physicians, and 65% would consider applying complementary therapies themselves (30% reported having used them on their patients). The study highlights that patients’ families showed interest in these types of therapies. A total of 6.5% of the pediatricians interviewed by Schlarb et al. [40] used homeopathy and naturopathy as therapeutic options for recurrent abdominal pain.

#### 3.6.4. Multidisciplinary Team

A management form highlighted by two studies is to have a multidisciplinary team to rely on [35,36] since, as shown by Glazebrook et al. [44], respondents found it unsatisfactory to treat children with medically unexplained diseases and demanded support; a fact that is not affected by sex but by profession. Such a need for support is greater with physicians than with nurses, which, in turn, is greater than that of other related professionals. Fifteen percent of the physicians surveyed by Bhatia et al. [36] mentioned that specialized pediatric centers with multidisciplinary teams (pediatricians, child psychologists, and physiotherapists) were the optimal clinical setting for the management of pediatric chronic pain. However, in the study by Miró et al. [35], only 6% of general practitioners and 24% of pediatricians were members of a multidisciplinary group. Moreover, 80% of respondents reported the absence of coordination between primary and specialized care.

In several analyses, it was possible to find that the patient with pediatric chronic pain is referred to other professionals with the aim of requesting complementary tests that shed light on the diagnosis [5,33,34,40,41,43], since they consider themselves to lack adequate material resources [36]. The studies by Schlarb et al. [40], Youssef et al. [34], and Høie et al. [5] request these tests from doctors, without indicating a specific specialty. The studies by Schurman et al. [33] and Edwards et al. [41] request the tests from gastrointestinal specialists. According to Koechlin et al. [43], the most frequently selected healthcare professionals seeking a different approach were rheumatologists, gastroenterologists, and hepatologists (49%), followed by neuropediatricians (47.3%) and orthopedists (40.7%).

The studies recommending the referral to another healthcare professional to intercede in the management of the child as a therapeutic option selected the following healthcare providers: gastrointestinal disorders physicians, nutritionists [39], pediatric and adult pain specialists, hematologists and oncologists [36,38], surgeons [36], neurologists [36], rheumatologists [36], physicians specialized in complementary therapies [37], physiotherapists and/or occupational therapists [43], and mental healthcare professionals (when the pain was assumed to have psychological bases) [33,40,41,43].

Thompson et al. [38], aiming to ask pediatricians which healthcare providers should assess and treat pediatric chronic pain, concluded that 32.3% of respondents believed it was the pediatricians’ sole responsibility, 15.8% believed that the responsibility should be shared with other specialists, and more than half thought it should be the specialist physicians’ sole responsibility (58.1% believed that a pediatric pain specialist should be responsible, 39.6% pediatric oncologists, 26.1% primary care centers, 6.3% adult pain specialists, and 2.3% believed they should be referred to emergency services).

### 3.7. Metacognition

Investigations conducted by Youssef et al. [34], Miro et al. [35], Riaño et al. [42], Sawni et al. [37], Koechlin et al. [43], and Al-Khotani et al. [45] assessed the self-perception of healthcare professionals regarding their pediatric chronic pain knowledge. Over 80% of those surveyed by Youssef et al. [34], Miro et al. [35], and Riaño et al. [42] deliberated that they did not have adequate knowledge about pediatric chronic pain (recurrent abdominal pain in the study by Youssef et al. [34]), with no significant differences by age group, sex, place of work [42], or years of work experience [34,35]. Recent data by Koechlin et al. [43] reported that 20% of the participants deliberated that they had a lot of experience in the management of pediatric chronic pain patients, compared to 41.3% of the participants who deliberated that they did not; in this case, the years of professional experience and male sex were associated with greater confidence in pediatric chronic pain treatment.

They also mentioned the inadequate training received [34,42,43], with 50% of the respondents in the analysis by Miro et al. [35] stating that they did not receive any sufficient training on pediatric chronic pain management during their academic studies, and that specific training was received in postgraduate courses and in continuing professional development according to 61.3% of the participants; these data match with those by Koechlin et al. [43], in which 78.5% reported they did not receive any specific training for pediatric chronic pain diagnosis and treatment. The need to increase specific training in pediatric chronic pain management emerges in the majority of these investigations [35,37,45].

## 4. Discussion

The results presented here may indicate that the current knowledge about pediatric chronic pain by healthcare professionals seems to be unsatisfactory, especially concerning its assessment and management. In terms of assessment, there is no consensus on which tool should be used; additionally, in relation to management, they focus on behavioral approaches and symptomatic and complementary therapies without considering the rehabilitative approach that would allow functional improvements in children suffering from pediatric chronic pain. The knowledge of health professionals is outdated and does not point to the latest research evidence, which highlights the need for training on this condition.

The articles included in this review revealed that individual factors such as race [37,38], age [35,40], and years of experience did not significantly affect the level of pediatric chronic pain knowledge [34,35,42,44], although Heinsch et al. [39] found more marked deficiencies in older generations. According to Al-Khotani et al. [45], there was also no difference between generalists and specialists (physicians and dentists according to their study). The analyses also revealed the absence of significant differences in the degree of knowledge about the healthcare professionals’ location [5,40,41,45]. However, gender did influence the attitude and management of healthcare professionals towards chronic pain in children. Women were more sensitive to the pain of their pediatric patients [42] and were more likely to use complementary therapies [37,38]. Male sex was also associated with a higher degree of confidence in pediatric chronic pain management [43], although this could be the result of a self-confidence bias [46]. This lack of effect of sociodemographic factors on the extent of pediatric chronic pain knowledge leads to the hypothesis that the problem could be associated with the complex nature of pediatric chronic pain and the difficulties related to its assessment and management [47], but also with the lack of contents dealing with pain in the curricula of all healthcare professionals [11,48,49,50,51,52]. This leads to the speculation that these curricula would need to be modified to improve both knowledge and attitudes of healthcare professionals towards pediatric chronic pain.

The reviewed studies mention the negative consequences of pediatric chronic pain, although no investigation highlights the predisposition to develop chronic pain in adulthood, as currently indicated [10]. Bhatia et al. [36] reported a fair or good prognosis for pediatric chronic pain in the majority of their respondents; these data do not match with those reported by Hassett et al. [53], who retrospectively interviewed a sample of 1045 adult patients with chronic pain and found that about 80% indicated a persistent pain from childhood to adulthood, thus suggesting an unfavorable prognosis. Neither did any study highlight the relationship between sleep and chronic pain in children, although it is known that sleep disturbance is significantly associated with an increase in pain intensity and pain interference [54]. Youssef et al. [34] stated that, although children with chronic pain showed a higher number of school absences, there was no associated comorbidity. This statement contrasts with data obtained in other recent investigations, where school absenteeism was shown to interfere with school functioning, lack of meeting with friends, inability to play sports, bullying, and harassment as well as teachers and peers’ mistreatment [55,56,57]. Therefore, pain seems to generate school absenteeism inducing a negative effect on the students’ participation in school activities. The effects of chronic pain on the health and general well-being of children and adolescents are becoming increasingly evident, and chronic pain in this population can negatively influence their physical, psychological, and social development.

When mentioning the pediatric chronic pain etiology, most of the surveyed agree that pain can be explained physically or psychosocially, giving more importance to the psychosocial component; although, there are studies in which respondents show a different attitude, stating that children can fake symptoms [34] without considering the severity of the disease [33,34]. This biopsychosocial model has been especially influential in the area of chronic pain. We now know that pain is modulated by many somatic, psychological, and social factors, but none of the articles in this review refer to the specific influence of catastrophizing and kinesiophobia [58] in contributing to the maintenance of pain and its increased intensity. The new paradigm of pain conceptualization, besides considering the various effects on its modulation, highlights that they also depend on the evaluative context of the noxious input [20]. This nociceptive input was already discussed by Melzack when he described the neuromatrix theory [59] and provided a conceptual framework for chronic pain, which would be produced by the output of a widely distributed neural network in the brain and not directly by the sensory input caused by an injury [60]. Hyperexcitability of the central nervous system is currently considered to be the most important cause in the development, persistence, and treatment of chronic pain [61,62], understanding central hyperexcitability as a form of maladaptive neuroplasticity which involves the brain producing pain even when there is no apparent somatic nociceptive signal [63,64]; however, none of the articles mention it. This leads to the belief that the surveys conducted are not up to date in assessing the level of knowledge about pediatric chronic pain.

On the other hand, according to the 2018 review by Pas et al. [10], evidence of central hyperexcitability among the pediatric population was found in children with juvenile rheumatoid arthritis, juvenile fibromyalgia, recurrent abdominal pain, and migraine. Some of these pediatric population groups have been identified in articles, such as recurrent abdominal pain and migraine; however, regarding diseases such as juvenile rheumatoid arthritis or juvenile fibromyalgia, there is no reference to them, so it could be said that this is also another factor potentially affecting underdiagnosis.

Regarding pediatric chronic pain assessment, there is currently no general consensus among health professionals on how to carry it out. It is striking that although almost all health professionals consider the assessment of pediatric chronic pain to be fundamental, no assessment method was known, and if they did know it, they did not usually apply it [42]. This aspect has been improving in some of the research on the knowledge of health professionals over time [35], and the tendency is to use assessment tools such as self-report by the patient and/or parents [38], subjective assessments of the child’s face or behavior, and the pediatrician’s examination [43], which seek to evaluate domains related to pain intensity in the majority of respondents [35,38,43] and side effects of treatment and satisfaction with the treatment used, with fatigue, sleep, and social function being less evaluated domains [35]. Regarding gastrointestinal disorders, it is should be noted that the pediatricians surveyed are unaware of and/or do not use the Rome criteria, since their use would facilitate a common language for the clinical diagnosis, management, and investigation of gastrointestinal disorders [65].

Although the majority of the articles reported here highlighted the multi-factorial nature of the pain, health professionals—during the assessment of the pain—do not consider the biopsychosocial approach, thus leaving important domains to be assessed. These are the physical function (e.g., mobility, fatigue, and quality of sleep), pain interference, psychological function, school function, and quality of life. In particular, it is known that improvement in quality of life is directly related to pain control and to the control of chronic pain regardless of its etiology and that this affects the quality of life scales [66]. Moreover, pain in children should be monitored not only in healthcare facilities, but also at school, home, and in the child’s environment. Therefore, parents, family members [67], and teachers [57] should also be included in the assessment. The current lack of use of specific evaluation tools by healthcare professionals might result in underdiagnosed pediatric chronic pain and, therefore, in undertreatment. This exacerbates its chronicity and the occurrence of high-impact chronic pain among children, a term used to identify children with significant levels of interference in life due to chronic pain [68].

The knowledge of healthcare professionals on pediatric chronic pain management is one of the most commonly evaluated variables [34,35,36,37,42,43,45]. Some incorrect beliefs have been overcome [69,70] and multiple studies showed an improvement in children’s pain management [71,72]. However, the participants in the studies reviewed here report that they did not receive sufficient training in the management of pediatric chronic pain, and there is a disparity of approaches, none of which appear to be effective. Recently, Peng et al. [73] stated that medical education worldwide lacks adequate training in pediatric pain management, as confirmed by previous research [21,71,74,75]. It should be kept in mind that the approach adopted by healthcare providers regarding pain management is significantly affected by their knowledge and attitudes towards pain; thus, if the knowledge is insufficient, the management will be inadequate.

The approaches covered in this review include behavioral approaches [33,39,40,41], symptomatic approaches [5,36,38,40,42], and complementary therapies [37]. Behavioral approaches include psychoeducation, relaxation, negative cognitions’ identification and treatment, values- and acceptance-based exercises, behavioral exposures, and parent training. Among symptomatic approaches, the pharmacological is selected by all authors mentioning this type of intervention [5,36,38,40,42], and physiotherapy is selected in only one study [40]. Mu et al. [76] review the effectiveness of non-pharmacological management in relieving chronic pain in children and adolescents, concluding that relaxation programs and bioremediation treatment could reduce recurrent headaches and pain intensity in children and adolescents in the short-term lasting up to six months. In addition, Wren et al. [77] and Revivo et al. [78] emphasize the need to shift from immediate analgesia to functional improvements in pain chronic management. This can be done with interventions based on physical exercise and within the rehabilitation scope. Specifically, they should aim to improve strength, flexibility, endurance, joint stability, weight-bearing tolerance, coordination, balance, and proprioception [78,79,80,81] and should prioritize active interventions over passive ones [47]. This statement, identifying that exercise is fundamental in rehabilitation for children and adolescents with pediatric chronic pain, was already defended by Clinch and Eccleston (2009) [82], although it is not mentioned in any of the studies reviewed here. Another approach not considered in the reviewed articles is child- and family-directed pain education. Pain education approaches shown to be effective have the use of a biopsychosocial model of chronic pain in common [83]. A clear explanation of this model allows for a reconceptualization of chronic pain, which, in turn, can produce positive effects that will lead to improvements in pain and disability outcomes from interventions [84]. Therefore, it is crucial that healthcare providers help their patients with chronic pain to learn more about their pain and understand their condition. The fact that neither physical activity nor education in pain neuroscience is contemplated in the reviewed articles confirms that the knowledge regarding pediatric chronic pain management is outdated and does not point to the latest research evidence.

The multidisciplinary approach to management in pediatric chronic pain with medically unexplained symptoms is also advocated in most of the research in this review [5,33,34,36,37,38,39,40,41,43,44] and supported by other authors [85,86]. However, our results also show that there is significant heterogeneity among pediatric pain managers. Several authors of this research consider the referral to other healthcare professionals necessary with the aim of a more extensive evaluation [5,33,34,38,41,44] or a different approach [36,43]; although they mention that this referral is often due to parental pressure and does not usually yield any somatic diagnosis [5,33]. Other studies in this review consider the referral to mental healthcare professionals as a necessity in the management of pediatric patients with chronic pain [33,40,41,43,44]. Another question that arises is who should be responsible for the child with pediatric chronic pain in a multidisciplinary team. It appears that primary care pediatricians are reluctant to treat pain and seek referral to another specialist [38]. This may be due to the relative rarity of severe cases among their patients, lack of training in pain management, or fear of medication side-effects [36,38,87,88]. In contrast, according to Stille et al. [89], the fact that the main responsibility for pediatric pain management lies with pain physicians could lead to difficult implementation due to the scarcity of these specialists, which hinders communication between them and other physicians, hence causing delays in patient treatment. However, Miro et al. [90] believed that team leaders could be from any discipline since personal features and willingness to be a leader are considered more important aspects than specific background or training. In the management of chronic pain, it is important to consider not only the multidisciplinary team, but also interdisciplinary care, which goes beyond referral to other professionals and is defined by the International Association for the Study of Pain as “multimodal treatment provided by a multidisciplinary team that collaborates in assessment and treatment using a shared biopsychosocial model and goals” [91].

By evaluating the articles published in the period 1994–2022, we noticed that there are constants that are preserved, such as the need for more training. In all articles, the healthcare professionals stressed that they had difficulties with the assessment and management of chronic pain in children. Concerning the assessment of chronic pain, most healthcare professionals, especially in the case of chronic abdominal pain, preferred to refer to other specialists in order to rule out organic factors. In the most recent articles, there is a need for the creation of multidisciplinary teams, although, in general, the absence of such teams is emphasized. In terms of management, the pharmacological option continues to be the most important. In the case of chronic abdominal pain, the treatment is especially aimed at offering peace of mind and comfort for the patient/family and improving their lifestyle. As for the causes of pain, misconceptions are being banished, and emphasis is given to psychosocial aspects. Currently, we continue to detect a lower prevalence than the evidence suggests and little awareness of the negative consequences of pediatric chronic pain on the life of children and their families.

### Strengths and Limitations

The main strength of this review is the broad and systematic search for articles dealing with this topic. Although there is a large number of studies dealing with healthcare professionals’ knowledge of pediatric pain, most of those published, up to date, are focused on post-surgical care, palliative care, or procedural pain, and those dealing with pediatric chronic pain knowledge are limited. Another strength is the inclusion of all healthcare personnel, considering that this type of patient approach is from an interdisciplinary perspective. One of the main limitations that should be considered when analyzing the findings is that there is no validated tool that assesses health professionals’ pediatric chronic pain knowledge, which makes it difficult to obtain common data that can be compared. Most of the studies used questionnaires specifically designed by the authors that had not been formally validated and of which no psychometric properties were reported, therefore, confidence in the results may be limited. Another limitation was that, although we wanted to include all healthcare professionals, almost all studies were focused on pediatricians and/or nurses. Most authors did not consider race among the sociodemographic characteristics of respondents, nor was it taken into account among children with chronic pain visited by healthcare professionals; if this variable had been collected, it could have been explored whether race affects respondents’ perception of chronic pain.

This review can provide guidance to healthcare training providers to justify the need to include training in the assessment and management of pediatric chronic pain among healthcare professionals.

## 5. Conclusions

Based on our findings, we believe that it is essential to improve the training of healthcare professionals with regard to pediatric chronic pain. In addition, we propose that the following steps be used as a guide when planning and implementing pain management. First of all, it is essential to frame chronic pain in children in a biopsychosocial context that facilitates the execution of interdisciplinary care. The assessment should not only focus on the symptoms (location, intensity) of pain, but also on the interference they have in the child’s life and in his or her family and school environment. Pain management should include education in pain neuroscience for the child and his or her environment as well as non-pharmacological therapies, giving special importance to physical activity. Treatment planning must be in harmony with the biopsychosocial framework. Furthermore, it is essential not only to perform effective treatment as soon as possible, but also to develop preventive programs that include the child, parents, and teachers.

## Figures and Tables

**Table 2:** Sociodemographic characteristics of the health professionals included in the clinical studies of the systematic review.

Author	Sample	Location	Sex		Age (Years)	Race or Ethnicity	Profession	Place of Work								Experience (Years)
			M	F				Public	Private	Hospital	Outpatient	Schools	Rural	Urban	Suburban	
**Edwards et al.** (1994) [41]	116	USA	73	41	30–>60	-	Pediatricians	-	-	-	-	-	-	27%	68%	
**Riaño et al.** (1998) [42]	157	Spain	81	73	<45–>45 (AA 41)	-	Pediatricians Pediatric Residents Pediatric Surgeons	100%	0%	49%	56%	0%	13%	87%	-	-
**Sawni et al.** (2007) [37]	648	USA	311	337	<35–>59	Caucasian: 81%; Asian 9%; African-American 4%; Hispanic 3%; Others 2.5%; Native American 0.5%	Pediatricians	46%	54%	30%	70%		-	-	-	<15: 55% >15: 45%
**Youssef et al.** (2007) [43]	141	USA	0	141	-	-	Nurses	-	-	-	-	-	-	-	-	26.2 YA
Bhatia et al. (2008) [36]	339	United Kingdom	-	-	-	-	Clinicians	-	-	-	-	-	-	-	-	-
**Glazebrook et al.** (2009) [44]	118	United Kingdom	37	81	-	-	Pediatricians Nurses Physiotherapists Occupational Therapists	-	-	-	-	-	-	-	-	9.72 YA
**Thompson et al.** (2010) [38]	303	USA	154	149	<40–>51	White, non-Hispanic: 55.8%; Black, non-Hispanic: 4.6%; Hispanic: 16.5%; Other 23.4%	Pediatricians	50.5%	76.2%	50.5%	73%	0%	-	-	-	0–10: 32.0% 11–20: 34.0% >21: 34.0%
**Schlarb et al.** (2011) [40]	167	Germany	67	100	35–70 (AA 51.9)	-	Pediatricians	-	-	-	-	-	-	-	-	21.4 YA
**Schurman et al.** (2014) [33]	470	USA	-	-	-	-	Pediatricians	-	-	-	-	-	17%	26%	56%	>30: 15% 11–30: 59% ≤10: 26%
Al-Khotani et al. (2015) [45]	248	Sweden and Saudi Arabia	124	124	26–52	-	Dentists Orofacial Specialists Maxillofacial Specialists Periodontists General Practitioners	-	-	-	-	-	-	-	-	-
**Høie et al.** (2017) [5]	17	Norway	0	17	29–65	-	Nurses					100%	-	-	-	
**Heinsch et al.** (2019) [39]	327	Australia New Zealand	130	197	<34–75	-	Pediatricians	71.3%	28.1%	48.9%%	50.5%	0%	-	-	-	
**Miro et al.** (2020) [35]	191	Spain	101	90	-	-	Pediatricians General Practitioners	-	-	-	-	-	-	-	-	21 YA (SD 8)
Koechlin et al. (2022) [43]	337	Switzerland	99	238	35–>65	-	Pediatricians			46.6%						>10 años 66%

Abbreviations: AA: Age Average; YA: Years on Average; SD: Standard Deviation.

**Table 3:** Questionnaires used in each clinical trial to assess the knowledge of the health professionals involved in the management of pediatric chronic pain.

Author	Pediatric Chronic Pain Type	Children’s Age	Questionnaire
Al-Khotani et al. (2015) [45]	Orofacial pain and temporomandibular disorders	-	Survey adapted from Le Resche et al. and Tegelberg et al., validated
Bhatia et al. (2008) [36]	General chronic pain	-	Compiled by the authors
Edwards et al. (1994) [41]	Recurring abdominal pain	-	Compiled by the authors (10 question items plus demographic data and a description of the recurring abdominal pain patient)
Glazebrook et al. (2009) [44]	Medically unexplained pain	-	24 item questionnaire compiled by the authors (validated)
Heinsch et al. (2019) [39]	Abdominal pain and gastrointestinal symptoms	0–16 years	Compiled by the authors
Høie et al. (2017) [5]	General chronic pain	13–16 years	Interviews that focused on the perceptions of nurses on the causes of pain and how adolescents express and manage daily pain.
Koechlin et al. (2022) [43]	General chronic pain	-	Compiled by the authors
Miro et al. (2020) [35]	General chronic pain	-	Own questionnaire modified from a previous one developed by Miro et al. (23 questions)
Riaño et al. (1998) [42]	General chronic pain	-	Compiled by the authors (95 questions)
Sawni et al. (2007) [37]	General chronic pain	-	27 Questions; questionnaire based on a survey used in a previous study (Sikland_1998)
Schlarb et al. (2011) [40]	Recurring abdominal pain	-	Compiled by the authors
Schurman et al. (2014) [33]	Recurring abdominal pain	-	The Pediatrician’s Gastroenterology Practice Survey (PGPS) adapted
Thompson et al. (2010) [38]	Severe chronic pain	-	Compiled by the authors
Youssef et al. (2007) [43]	Recurring abdominal pain	-	Compiled by the authors (21 items)

**Table 4:** Summary of the pediatric chronic pain management.

Author	Behavioral Treatment	Treatment Symptomatic	Complementary Therapies	Multidisciplinary Team
**Al-Khotani et al.** (2015) [45]	- Not provided	- Not provided	Not provided	- Not provided
Bhatia et al. (2008) [36]	- Cognitive therapy: 17.7%	- Medication: 41.3% - Physiotherapy: 23.6% - Nerve stimulation techniques (TENS): 17.7% - Nerve blocks: 1.2% - Most pain physicians use a combination of three or more modalities.	Not provided	83% of pain physicians and 55% of general practitioners referred patients to the hospital - Pediatricians: 79% - Orthopedic surgeons: 70% - General practitioners: 58% - Oncologists: 13% - Neurologists: 12% - Pediatric surgeons: 11% - 15% of pain physicians specifically mentioned that specialized pediatric centers with multidisciplinary teams (pediatricians, child psychologists, and physical therapists) are the optimal clinical
Edwards et al. (1994) [41]	- Inform parents that everything is normal: always (SD 0.4) - Reassure parents and child that there is no illness: always (SD 1.1) - Raise the possibility that emotions or psychological factors are playing a role: always (SD 1.1)	- Medication: sometimes (SD 1.8)	Not provided	- Refer to GI specialist: rarely (SD 1.4) - Refer to MHP: rarely (SD 1.5) - Refer to psychologist: rarely (SD 1.4) - Refer to psychiatrist: rarely (SD 1.4)
Glazebrook et al. (2009) [44]	Not provided	Not provided	Not provided	- Physicians perceived a greater need for support than nurses Nurses perceived a greater need for support than medicine related professionals
Heinsch et al. (2019) [39]	- Physician recommendations that - consistent with a cognitive behavioral treatment for pain: 29.8% Written information: 47.4%	- Anti-diarrhoeal agents (loperamide): 11.1% - Metronidazole (empiric course): 9% Probiotics: 34.6%	- Dietary modifications suggested by the physician: 34.3% Peppermint oil: 17%	Refer to a paediatric gastroenterologist: 30.5% Refer to a dietitian: 46% Refer to a psychologist or counsellor: 15.9% Referral for hypnotherapy: 2.1%
Høie et al. (2017) [5]	Not provided	All the nurses stated that adolescents consumed painkillers such as paracetamol.	Not provided	Not provided
Koechlin et al. (2022) [43]	Not provided	Not provided	Many participants had also referred patients to hypnotists, acupuncturists, and practitioners of alternative medicine.	- Refer to another specialist: 75% - Refer to a service specializing in pain in children and adolescents: 90% - The most frequently referred specialists were: Gastroenterology and hepatology: 35.3% Neuropediatrics: 34.12% Orthopaedics: 29.4% Also to non-pediatric pain specialists (internal medicine, rheumatology, neurology, psychiatry, and anesthesia.
Miro et al. (2020) [35]	Not provided	Not provided	Not provided	- 6% of general practitioners and 24% of pediatricians were members of a multidisciplinary group. 85% reported that there was no coordination between primary and specialized care.
Riaño et al. (1998) [42]	Not provided	Medication: - Acetylsalicylic acid: 12% - Paracetamol: 18% - Paracetamol-codeine: 37% - Metamizole (dipyrone): 28% - Other NSAISD: 24% - Morphine or other opioiSD: 30% Tricyclic antidepressants: 3%	Not provided	Not provided
Sawni et al. (2007) [37]	Not provided	Not provided	96% believe their patients are using some form of CAM 30% reported using CAM therapies on their patients. The most common CAM therapies were: - Prayer for healing: 9% - Relaxation: 9% - Massage therapies: 8% - Imagery: 6% Herbs, megavitamins, lifestyle diet, biofeedback: 5%	Not provided
Schlarb et al. (2011) [40]	- Counseling, Support and Reassurance: 20.1% - Psychotherapy: 3.8% - Behavioral Interventions: 3.3% Relaxation: 2%	- Medication and physical therapy: 11.1% Treatment for Constipation: 2.6%	6.5% Homeopathy, Naturopathy	- 39.6% Request psychological treatment in children with recurrent abdominal pain. 2% Referral to Medical Specialist
Schurman et al. (2014) [33]	- 74% Change of Lifestyle. - 61% Peace of Mind. - 51% Positive Coping. - 26% Interventions at School. - 12% Biofeedback Referral. 2% Dairy.	- Laxatives: 30% - Selective serotonin reuptake inhibitor: 6% - AntaciSD: 6% - Imipramine, Elavil: 4% Probiotics: 4%	- 53% Fiber Supplementation. 6% Herbal Supplements.	- 54% Gastrointestinal specialist referral. 80% Family Request 70% Testing 60% Pain does not Decrease 50% Pain Increases 60% Uncertainty in Treatment 5% Routine Referral 2% Red Flag Symptoms 1% other - 50% Mental Health Practitioners referral. 80% consider pain to have a psychological basis. 50% family request. 20% testing. 30% pain does not decrease or increases. 30% uncertainty in treatment. 2% routine referral. 4% request for collaboration. 3% unimportant findings by the GI specialist. 2% psychological symptoms: 8% never or rarely 1% other
Thompson et al. (2010) [38]	Not provided	Medication - NSAISD almost always: 66.9% - Acetaminophen almost always: 61.7% - Intermittent opioiSD: 38.1% - Permanent opioiSD: 25.4% - Patient-centered analgesia: 17.7% Massage almost always: 9.5% - Acupuncture almost always: 4.6%	Not provided	The professional responsible for the child in pain should be: - 32.3%: pediatricians - 15.8% Other professionals besides him/herself. - 58.1% Pediatric pain specialist. - 39.6%: Other specialist (pediatric hematologist or oncologist) - 26.1%: Local hospice - 6.3%: Adult pain specialist 2.3%: Local emergency services
Youssef et al. (2007) [34]	Not provided	Not provided	Not provided	- 84% believed that communication with physicians about RAP is poor - 70% of the respondents believed that a more extensive evaluation by the physician was needed

## Supplementary Materials

The following supporting information can be downloaded at: https://www.mdpi.com/article/10.3390/children10040665/s1, Table S1: Characteristics of the studies included in the systematic review.
