# Supplementary material for: Healthcare Professionals’ Knowledge about Pediatric Chronic Pain: A Systematic Review
Source: Children (Basel). 2023 Mar 31;10(4):665. doi: 10.3390/children10040665 (PMC10136554; doi:10.3390/children10040665)
Supplement: Supplementary file 1 [file children-10-00665-s001.zip › children-2283505-supplementary.pdf]

Supplementary Table S1. Characteristics of the studies included in the systematic review.

Author	Knowledge	Self-perception	Etiology	Evaluation	Consequences
Al-Khotani et al. (2015) [45]	- OFP and TMD specialists in Sweden supported a consensus of 78% of responses: 71% in chronic pain and pain behavior, 89% in the Etiology domain, 64% in Diagnosis and Classification, and 100% in Treatment and Prognosis.	There is a general agreement over the need to increase the level of professional knowledge about OFP / TMD in children and adolescents.	- Swedish pediatricians differed least from the reference group, with only one of the five responses significantly different.<br>- There was disagreement between all groups compared with the reference group on all other statements in this domain.	Maxillofacial surgeons in Sweden showed less difference compared to the reference group. The groups in Arabia showed greater difference.	
Bhatia et al. (2008) [36]	Most physicians reported that they lacked training in the management of pediatric chronic pain. Of the 23% of pain physicians who had received training in pediatric pain management, only 8% agreed that the training had been adequate.				- Functional disability: school absence (most respondents agreed), reduced socialization and inability to play sports, and sleep interruption.<br>- Family disruption.<br><br>75% believe that pediatric chronic pain has a fair or good prognosis. Poor prognosis was reported by 5% of respondents.
Edwarsd et al. (1994) [41]			- Multiple organic, personality, and environmental factors: almost always.	**1st Visit**:<br>- Medical history: always<br>- Physical examination: always<br>- Psychosocial history: always<br>- Urinalysis: always	

			- Organic, psychogenic, and unknown causes: almost always.<br>- Organic and psychogenic causes: sometimes.<br>- Unidentified organic cause: sometimes.	- Complete blood test: sometimes<br>- Rectal examination: sometimes<br>- Stool culture: sometimes<br>- Urine culture: sometimes<br>- Stool for egg and parasites: sometimes<br>- Erythrocyte sedimentation rate: sometimes<br>**2nd Visit:**<br>- Psychosocial history: always<br>- Contact with school: sometimes<br>- Abdominal X-ray: rarely<br>- Upper GI series: rarely<br>- Abdominal/ pelvic US: rarely<br>- Barium enema: rarely<br>- Intravenous pyelogram: rarely	
Glazebrook et al. (2009) [44]		- Professionals with more experience in working with children with MUS perceived it to be more demanding and less satisfying and perceived more need for support.<br>- Physicians found caring for children with MUS less satisfying than nurses (mean difference 1.86, 95% CI, 0.8 to 2.9, p = 0.001) and medicine related professions (mean	Participants placed a strong emphasis on the psycho-social aspects of the syndrome, in particular, the role of the family in maintaining the condition.		

		difference 3.4, 95% CI, 4.98 to 1.89, p <0.001).<br>- Nurses were less satisfied caring for children with MUS than medicine related professionals (mean difference 1.58, 95% CI, 3.05, a) 0.11, p = .036).			
Heinsch et al. (2019) [39]			66% believed that FAP was a psychological problem.<br>8% believed that IBS was a psychological problem.	- 37.2% correctly identified Rome III criteria.<br>- 57.6% were unaware of Rome criteria.<br>- The use of Rome III criteria was only associated with outpatient setting.	- 82.8% believed that the quality of life for children with FAP is just as bad as that of children with IBS.
Høie et al. (2017) [5]			- High academic expectations and low stress tolerance.<br>- Reflection of difficult relationships and traumatic experiences.<br>- Reflection of an unhealthy lifestyle.<br>- Relationship to how parents deal with pain		- Teenagers are less emotionally strong now than they used to be.<br>- Much more school absenteeism.<br>- A lot more complaining.
Koechlin et al. (2022) [43]	Most of the participants stated that they had not received specific training in pediatric chronic pain management.	20% of respondents reported having a lot of experience and feeling confident in handling the pediatric chronic pain			

		(men felt more confident than women).			
Miro et al. (2020) [35]	50% reported that they had not been given any training in pediatric chronic pain management during their university studies. Most participants received specific training on pediatric chronic pain management in professional courses and postgraduate training.	More than 80% of the participants recognized significant gaps in their pediatric chronic pain training and were interested in improving.		84.8% of the participants stated that they evaluated pediatric chronic pain. The most frequently evaluated domains were pain intensity (80%), treatment side effects (67%), physical, emotional and social function of the child (69%), and overall satisfaction with treatment (60%). The least evaluated domains were fatigue (42%), sleep (61%), and social function (60%). The reasons for not doing pediatric chronic pain assessment were lack of time and the absence of an assessment protocol.	
Riaño et al. (1998) [42]	They lack knowledge of pain management, especially pediatric chronic pain management.	- 88% consider that they do not have adequate knowledge of pediatric chronic pain management.<br>- 18.5% demand support to improve their training in the assessment and management of pediatric pain.		- 35% are familiar with some pain assessment method.<br>- 24% of pediatricians over 45 years old said they were familiar with assessment methods compared to 40% of the younger group ($p < 0.05$).<br>- 82% have never applied any pain assessment method.	
Sawni et al. (2007) [37]	-18% had CAM formal knowledge and 55% were self-taught.	84% want more CAM courses.			

	- 49% report CAM personal use.				
Schlarb et al. (2011) [40]				35.6% perform tests to exclude organic causes and rule out allergies.	Situation that represents a source of stress and tension for all close family members.<br>- Moderate stress<br>-Noticeable stress: parents with RAP.<br>- Notable stress<br>-Intense stress for parents with IBD.
Schurman et al. (2014) [33]			Cause of RAP:<br>- 47% Constipation<br>- 27% Functional Abdominal Pain<br>- 17% Stress/Anxiety<br>- 11% General Psychological Causes<br>- 10% Specific Disorders<br>- 3% Diet<br>- 3% Other<br><br>Cause of FGID:<br>- 73% lack of organic etiology.	- 58% were not aware of Rome criteria and 7% used it in their practice.<br>- Blood tests: 74%.<br>- Urinalysis: 73%.<br>- Stool Culture: 41%.<br>- Abdominal Ultrasound: 27%.<br>- Psychological Consultation: 26%.<br>- Abdominal CT Scan: 4%<br>- Daily Diary: 2%	9% thought that RAP generated a functional disability that interfered with their life.
Thompson et al. (2010) [38]				- 84.2%: often or always use the patient's self-report.<br>- 87.1%: often or always use parents report.<br>- 66.7%: often or always use non-verbal scales.	

				- 55.5%: pain scales<br>- 49.5%: often or always use the pain diary.	
Youssef et al. (2007) [34]	In relation to medication:<br>- Good knowledge: 12% in those with <10 years of experience and 10% in those with >10 years of experience.<br>- Some knowledge: 75% in those with <10 years of experience and 64% in those with >10 years of experience.<br>- No knowledge: 12% in those with <10 years of experience and 14% in those with >10 years of experience.	Knowledge:<br>- 50% believed they had good knowledge of RAP<br>- 81% believed they lacked education about RAP<br>RAP Training:<br>- Lack of training: 77% in those with <10 years of experience and 88% in those with >10 years of experience<br>- Enough training: 20% in those with <10 years of experience and 9% in those with >10 years of experience.<br>- Too much training: 0%	- 3.6% believed that RAP is a disease.<br>- 31% did not know if RAP is a serious or minor condition.<br>- 47% thought they were faking it<br>- 61% thought they were seeking attention<br>- 31% thought it was nerves<br>- 29% thought it was sadness<br>- 78% thought they were lazy<br>- 25% thought they needed medication<br>- 52% thought they should relax more	More than 70% thought that an extensive medical evaluation was needed to rule out everything possible.	50% estimated that children with RAP miss more school than other children; school nurses do not seem to appreciate comorbidity associated with school absenteeism.
